# Practical consensus guidelines for the management of enuresis

**DOI:** 10.1007/s00431-012-1687-7

**Published:** 2012-02-24

**Authors:** Johan Vande Walle, Soren Rittig, Stuart Bauer, Paul Eggert, Daniela Marschall-Kehrel, Serdar Tekgul

**Affiliations:** 1 Pediatric Nephrology Unit, Ghent University Hospital, Ghent, Belgium; 2 Department of Pediatrics, Aarhus University Hospital, Skejby, Denmark; 3 Department of Urology, Children’s Hospital, Harvard Medical School, Boston, MA USA; 4 University Children’s Hospital, Kiel, Germany; 5 Urology Consultancy, Frankfurt/Main, Germany; 6 Department of Urology, Hacettepe University School of Medicine, Ankara, Turkey; 7 Pediatric Nephrology UZ Ghent, De Pintelaan 185, 9000 Ghent, Belgium

**Keywords:** Enuresis, Monosymptomatic, Consensus guidelines, Primary care, Management

## Abstract

Despite the high prevalence of enuresis, the professional training of doctors in the evaluation and management of this condition is often minimal and/or inconsistent. Therefore, patient care is neither optimal nor efficient, which can have a profound impact on affected children and their families. Once comprehensive history taking and evaluation has eliminated daytime symptoms or comorbidities, monosymptomatic enuresis can be managed efficaciously in the majority of patients. Non-monosymptomatic enuresis is often a more complex condition; these patients may benefit from referral to specialty care centers. We outline two alternative strategies to determine the most appropriate course of care. The first is a basic assessment covering only the essential components of diagnostic investigation which can be carried out in one office visit. The second strategy includes several additional evaluations including completion of a voiding diary, which requires extra time during the initial consultation and two office visits before treatment or specialty referral is provided. This should yield greater success than first-line treatment. *Conclusion:* This guideline, endorsed by major international pediatric urology and nephrology societies, aims to equip a general pediatric practice in both primary and secondary care with simple yet comprehensive guidelines and practical tools (i.e., checklists, diary templates, and quick-reference flowcharts) for complete evaluation and successful treatment of enuresis.

## Introduction

Evaluation and management of enuresis, a common condition, is not a priority in training programs for medical doctors (MDs), despite being a common condition. Frequent bedwetting (defined as >3 wet nights per week) has an estimated prevalence of nearly 10% in children aged 7 years [9, 16, 17, 22, 57] and is associated with embarrassment and significant emotional and practical difficulties for affected children and their families. Information available to families on the internet is often inaccurate, while guidelines for doctors are often center- or subdiscipline-dependent and are published in highly specific specialty journals not available to the majority of MDs in primary or secondary pediatric care [13, 18, 24, 33, 37, 41, 46, 47]. No unifying set of recommendations for a diagnostic and management algorithm of enuresis currently exists.

## Aims of this paper

This manuscript aims to present an international consensus on a practical, rational approach to the diagnosis and management of bedwetting in the primary care setting. Recommendations are based on the International Children’s Continence Society (ICCS) standardization document on monosymptomatic enuresis (MNE) [31], empirical evidence, and discussions of leading experts in pediatric urology and nephrology during a consensus meeting in 2009. The recommendations have been reviewed and endorsed by committees representing the American Academy of Pediatrics, European Society for Paediatric Urology (ESPU), European Society for Paediatric Nephrology, and the ICCS.

This guideline is intended as a practical supplement to the recent ICCS standardization report [31] and provides direction and tools (e.g., checklists, flowcharts, and diaries) for MDs to use in children with enuresis and their parents, by outlining minimum evaluation criteria, initial treatment options, and indications for referral to a specialist center/doctor.

## Background

**Table:** 

Box 1: Terminology
Definitions
*Comorbidity factors*: Factors proven to be associated with increased incidence of enuresis and/or increased therapy resistance
*Expected bladder capacity* (EBC): Calculated as [30 + (age in years × 30)] in milliliters
*Maximum voided volume* (MVV*)*: The largest volume of urine voided in a 24-h period, as documented in a bladder diary kept over 3–4 days, excluding first morning voids
Conditions
*Enuresis*: Intermittent incontinence while asleep in a child >5 years of age
*Monosymptomatic enuresis*: Enuresis with no other lower urinary tract symptoms
*Nocturnal polyuria* (NP): Overproduction of urine at night, defined as nocturnal urine output exceeding 130% of EBC for age
*Non-monosymptomatic enuresis*: Enuresis with other, mainly daytime, lower urinary tract symptoms
*Overactive bladder* (OAB): all children with complaints of urgency and frequency with or without incontinence
A full glossary of all relevant terminology and definitions can be found in the 2006 ICCS standardization paper [33]

The pathophysiology of enuresis is complex, involving the central nervous system (several neurotransmitters and receptors), circadian rhythm (sleep and diuresis), and bladder function derangements. A simplified screening process enables identification of two archetypes of enuresis:

- Underlying NP associated with low overnight vasopressin levels [38], decreased urinary osmolality, and poor likelihood of a desmopressin response
- “Small for age” bladder volume associated with OAB (overlaps with subtype of non-monosymptomatic enuresis (NMNE)—patients with lower urinary tract symptoms), reduced desmopressin response, and higher rates of response to the enuresis alarm

A combination of both forms is possible, and such patients generally respond well to combined therapy with desmopressin and an alarm. In addition to these urinary/bladder storage characteristics, all children with enuresis experience impaired arousal from sleep which prevents waking to void in the toilet [25, 32].

In children aged ≥5 years, enuresis is considered abnormal. Reasons for proactive management include the distress caused to child and family, difficulty of “sleeping over” on holiday or at friends’ houses, social withdrawal, reduced self-esteem [12], and potential disturbance of the child’s and the parents’ sleep architecture that may have an impact on daytime functioning and health [5, 7, 23, 60]. Moreover, untreated enuresis (especially if severe) can persist indefinitely, with prevalence rates in adulthood of 2–3% [61, 63]. As such, prompt therapy may avoid serious consequences for the child’s well-being. Additional reasons include the risk that some parents may be intolerant of their child’s wetting [3, 43, 55] and the significant inconvenience and costs associated with frequent laundering of bedsheets and clothing [34, 44]. The lack of public awareness that this is a treatable condition means that some families prefer to keep it a secret, hoping for spontaneous resolution, rather than seeking medical help [2].

Children who wet the bed, especially those with severe enuresis, are likely to benefit from appropriately timed treatment; the 2–3% who continues to wet into early adulthood without treatment may be prevented from enduring the prolonged impact of the incontinence [20, 27, 30, 34, 45].

## Management of enuresis

This clinical management tool (CMT) should help to:

- Raise awareness
- Provide current information to affected families and MDs
- Override false advice and misinformation from unregulated sources
- Limit recommendations to evidence-based treatment strategies

In primary care, “trial and error” treatment for enuresis is often the rule rather than the exception; this approach is a waste of time and money and increases frustration among families and doctors. It may also have an adverse psychological effect on the child [48]. We believe the rational therapeutic approach outlined in the ICCS standardization document [31] would lead to higher success rates.

Specialists have more time to perform a full diagnostic evaluation that may result in improved outcomes than primary care physicians, due to restrictions in time and budget that limit protocol adherence in every affected child. We also recognize that there may be country- and center-specific variations in the level of investigation possible. Therefore, we propose two levels of evaluation, allowing MDs to choose an appropriate strategy.

**Table:** 

Box 2: Strategies for evaluating patients with enuresis in primary care
*Strategy 1*: Minimal evaluation, covering only the essential components of diagnostic investigation during a single office visit that includes evaluation and treatment or referral to a consultant or specialty center
*Strategy 2*: All components of strategy 1 as well as a small number of additional evaluations that may require extra time during the initial consultation and two office visits before treatment or referral is provided. This probably carries a greater chance of successful first-line treatment

**Fig. 1:**
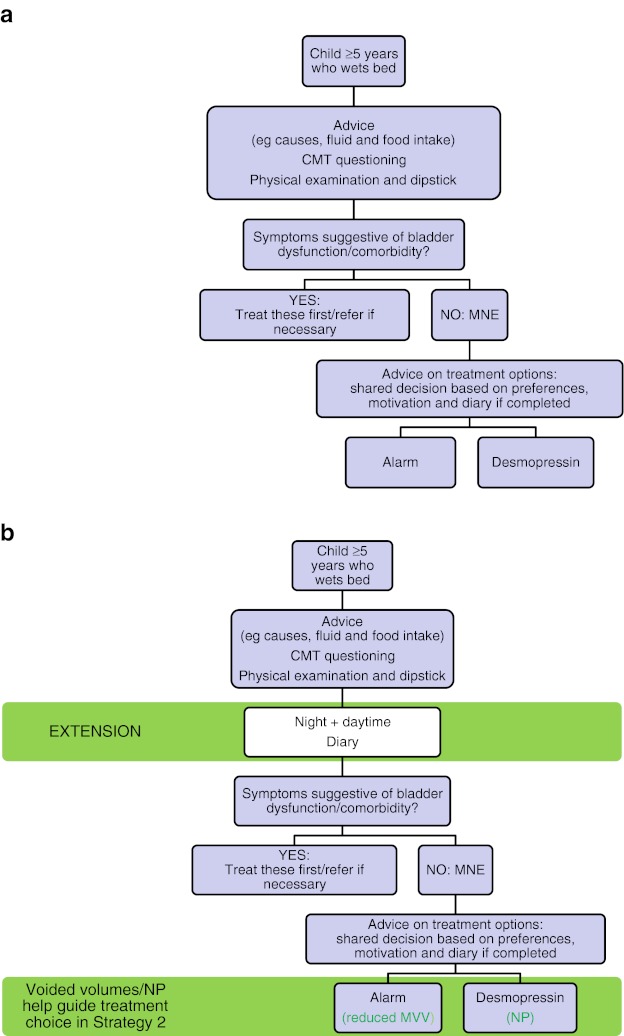
Flowcharts summarizing recommended evaluation of children with enuresis. **a** Strategy 1 = minimal; **b** Strategy 2 = optimal. *CMT* clinical management tool, *MNE* monosymptomatic enuresis, *MVV* maximum voided volume, *NP* nocturnal polyuria

If the chosen strategy is not successful, the child should be referred to a specialist who can provide a more in-depth evaluation of the problem. Flowcharts summarizing the recommended evaluation of patients with enuresis (strategies 1 and 2) are shown in Fig. 1.

## Basic evaluation process

### History taking—for strategies 1 and 2

Thorough history taking is an essential component for strategies 1 and 2. A checklist CMT, with footnotes explaining the relevance of each area of questioning and consequences for potential referral, is provided (Table 1) to assist with this process. Essential areas of questioning are listed with more detailed questions to elicit further information when necessary. Although the CMT is designed to be completed by the doctor, it could be adapted as a “self-assessment” test for patients/parents and given to families beforehand to reduce consultation time [11, 15].

**Table 1:**
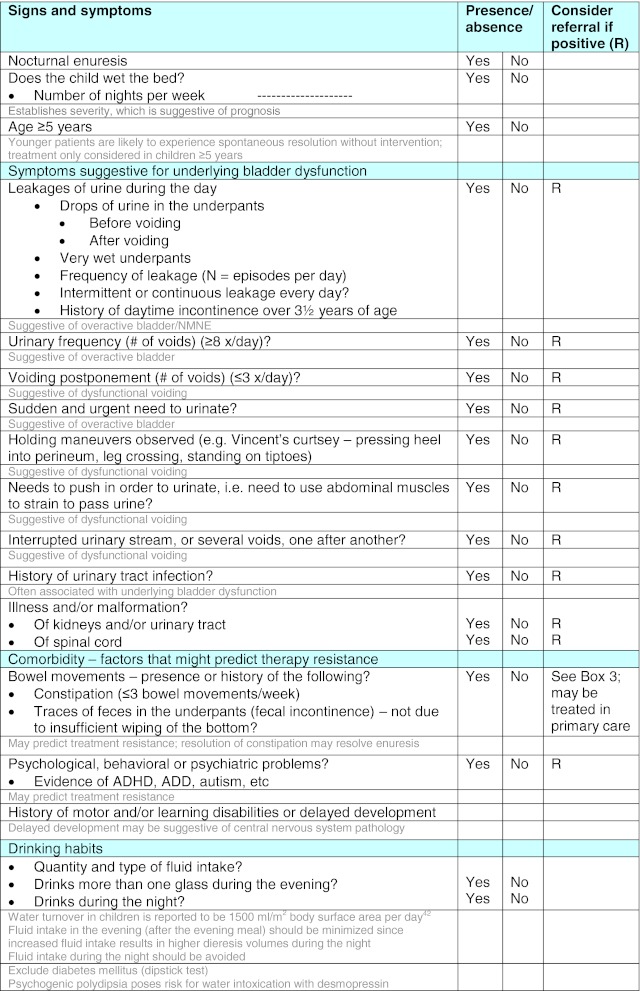
Checklist clinical management tool (CMT)

Notes for physicians are given in gray text

### Physical examination

A brief but thorough physical examination should be performed primarily to identify rare underlying anatomical (phimosis and labial agglutination) or neurogenic (spinal malfunction) causes. Relevant observations are listed in Table 2. Use of urodynamic studies, X-ray, and cystoscopy are not appropriate in this setting; if indicated based on data collected, the family should be referred to a specialist.

**Table 2:** Important physical examination and urinary test findings in children with enuresis

Strategy	Observation/test	To check for
1 (minimal)	Body weight and height	Growth retardation and/or failure to thrive suggestive of an underlying disorder
1 (minimal)	Genital examination—including examination of underwear	Physical abnormalities: hypospadias, phimosis, labial agglutination. Signs of fecal incontinence
1 (minimal)	Inspection of lumbosacral spine	Occult spinal dysraphism: dimple, lipoma, hypertrichosis, or sacral agenesis
1 (minimal)	Urine dipstick test	Glycosuria, infection (leucocyturia, nitrite test), hematuria, and proteinuria
2 (extended evaluation)	Rectal palpation if acceptable for child, parent/care giver	Fecal masses; left bowel palpation may be more acceptable to family
2 (extended evaluation)	Neurological examination (size, tight heel cords, hammer, or claw toes)	Signs and symptoms suggestive of lower spinal cord dysfunction
	The neurologic exam should include assessment of posturing with a stress gait or mirror movements	CNS abnormalities suggestive of a central cause

*CNS* central nervous system

**Table 3:** Expected age-related bladder capacity and interpretation of maximum and total voided volume overnight (all in milliliters), EBC calculated as: [30 + (age in years × 30)] in milliliters

Age (years)	BC (ml)	MVV below listed volume = reduced bladder capacity; consider alarm	Total volume below listed value = nocturnal polyuria; consider desmopressin
5	180	117	234
6	210	137	273
7	240	156	312
8	270	176	351
9	300	195	390
10	330	215	429
11	360	234	468
12	390	254	507
13	390	254	507
14	390	254	507
15	390	254	507
16	390	254	507
17	390	254	507
18	390	254	507

A pronounced nocturnal arginine vasopressin deficiency is seen in desmopressin responders only on nights with enuresis—therefore, NP should only be looked for on wet nights [41]. Excessive nocturnal urinary volumes indicated by diary data and various additional signs are suggestive of underlying NP [43, 62], e.g., absorbent underpants totally soaked overnight, urine soaking through to the bedsheets, multiple episodes of wetting in one night, early wetting in the first third of the night, a large volume of urine at the first void in the morning despite wetting overnight, a low daytime fluid intake followed by the majority of the intake in the late afternoon and evening

*MVV* maximum voided volume, *EBC* expected bladder capacity, *NP* nocturnal polyuria

### Patients with explicit NMNE

A consensus opinion advocates that daytime symptoms be treated before the enuresis is addressed as coexisting bladder dysfunction is associated with less favorable outcomes for both desmopressin and alarm therapy. Specialist referral is almost mandatory. Children with these findings are considered complex, and their management is controversial and outside the framework of this paper since little evidence-based medicine to guide treatment exists. Furthermore, therapy resistance is likely, and many of the proposed therapies require off-label use of medication.

### Patients with constipation

Enuresis (especially NMNE) often occurs concomitantly with constipation. Constipation should therefore be identified and treated before managing enuresis (see Box 3—the Rome criteria).

**Table:** 

Box 3: Rome III criteria for diagnosis of constipation [35]
At least two of the following criteria must be met for ≥2 months before diagnosis:
(a) ≤2 defecations in the toilet per week
(b) ≥1 episode of fecal incontinence per week
(c) History of retentive posturing or excessive volitional stool retention
(d) History of painful or hard bowel movements
(e) Presence of a large fecal mass in the rectum
(f) History of large diameter stools that may obstruct the toilet
Patients should not have a diagnosis of irritable bowel syndrome

### Other comorbidities

Eating and drinking habits should be reviewed. Helpful advice based on opinion rather than empirical evidence includes avoiding excessive fluids in the evening [24], avoiding caffeinated beverages (expert opinion), ensuring adequate fluid intake during the daytime, avoiding a high protein diet or salt in the evening (as these induce solute diuresis) [53], and remembering to void before bedtime.

### Discussion and reassurance

Paramount to the success of this process is an explanation at the outset of why these measures can be helpful. Children and families should be educated about the condition and reassured that (a) it is a common problem that they should not be embarrassed, (b) it likely affects other members of their peer group, and (c) there are effective treatments to resolve it. Frank discussion should be encouraged and educational materials provided for children and parents to help engage the whole family in the treatment process. Empathetic support should be provided if enuresis persists.

## Additional evaluations for strategy 2

Patient recall does not always correspond with actual voiding patterns. Where possible, it is ideal to provide families of child with no apparent symptoms of NMNE with bladder diaries (see template) for completion before a second appointment (evidence level 3, grade B). This has the advantage of helping to distinguish MNE from NMNE and to give important information on bladder capacity and nocturnal urine production.

Diary recommendations:

1. Daytime diary used to assess the child’s bladder capacity (see Template 1). Measurement of MVV (excluding the first morning void) needs to be made over a minimum of 3–4 days for accuracy; weekends or school holidays are ideal [21]. Any leakage of urine during the day as well as fluid intake volumes should also be recorded. The relevance of fluid intake volumes for treatment/advice is not proven, but is included to ensure maximum usability.

**Template 1:**
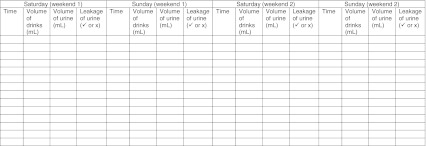
Daytime diary over two consecutive weekends

From this information, the most important observation for the clinician is the maximum voided volume. The largest urinated volume should be identified. It should be considered abnormally small or large if found to be <65% or >150% of expected bladder capacity (EBC), respectively [6] – see Table 2 listing EBC, reduced MVV, large MVV and NP values for 5–18 years
2. Bedwetting diary completed for seven consecutive days/nights (Template 2) to assess for the presence of NP. The volume of first morning void (in milliliters) must be added to the difference in diaper weight to calculate nighttime urine production. In patients with nocturia, the volume of nighttime voids should be added; (see Box 1 and Table 3 for values by age). NP should only be anticipated on wet nights [39].
  Bowel movements should be recorded to provide additional information regarding presence of constipation.

**Template 2:**
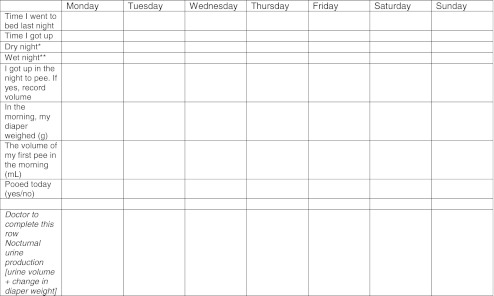
Overnight diary for seven consecutive nights

^a^Dry night is when you do not wet your bed or your diaper

^b^Wet night is when you wet your bed or your diaper

Families should be provided with a vessel for collecting and measuring urine volumes (e.g., 20 ml increments), and, if needed, diapers or pull-ups which can be weighed. The importance of completing both diaries for ascertaining the cause and likely most successful treatment should be explained.

Although no randomized studies have compared the outcome of strategies 1 and 2, there is significant evidence of the predictive value of both MVV and NP for outcomes with most common treatment modalities.

## Guidance for using enuresis therapies

### Monosymptomatic enuresis

**Fig. 2:**
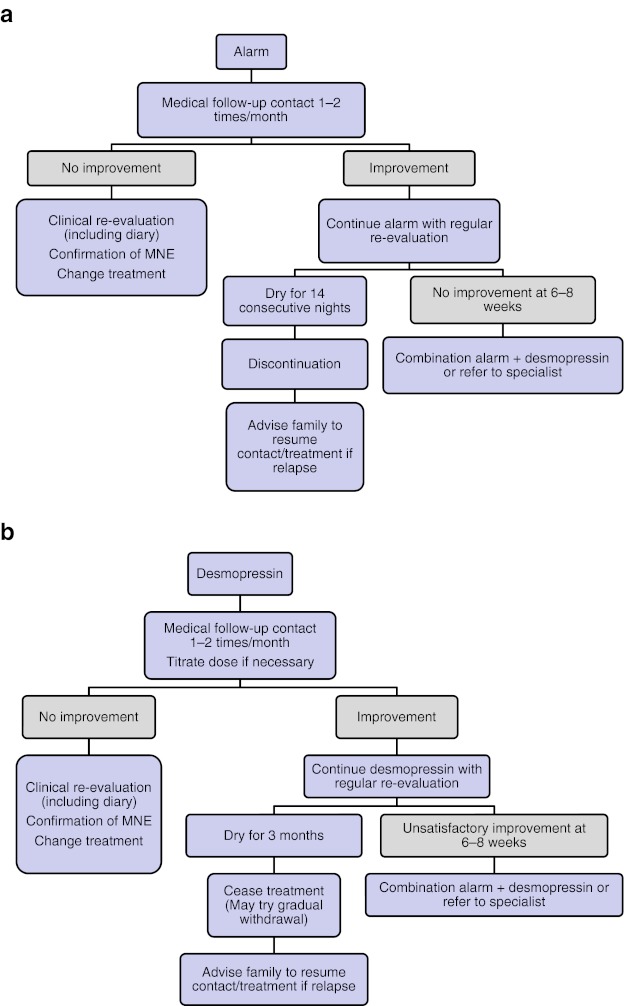
First-line treatment options for monosymptomatic enuresis (**a** alarm and **b** desmopressin)

MNE can be effectively treated by a general practitioner (Fig. 2). Two first-line treatment options are available—desmopressin and enuresis alarm. Their initial selection should be guided by the family’s level of motivation and their preference (strategy 1). Information from diaries (strategy 2) will identify one of four subtypes of MNE and allow further fine-tuning of treatment according to the child’s characteristics and family motivation.

1. Children with a normal urine output during the night and normal bladder capacity can be given either the alarm or desmopressin.
2. Children with smaller than expected bladder capacity for age will likely be desmopressin-resistant and more sensitive to the alarm.
3. Children with NP and normal bladder volume will be more sensitive to desmopressin.
4. Children with both excessive urine output and reduced bladder capacity may find combined therapy of alarm and desmopressin to be successful [26]. This strategy lessens the burden of alarm treatment as the alarm is triggered several times per night.

## Desmopressin therapy

Desmopressin is a synthetic analog of arginine vasopressin, the naturally occurring antidiuretic hormone. One of its major actions is to reduce the volume of urine produced overnight to within normal limits. Desmopressin has a level 1, grade A recommendation from the International Consultation on Incontinence (ICI) in 2009 [46]. It is available as a tablet (dosage, 0.2–0.6 mg) or a fast-melting oral lyophilisate (Melt; dosage, 120–360 μg). The latter is a recommended formulation for all children [6] and is preferred by children under 12 years [28]. It is not affected by nasal congestion or gastrointestinal transit and does not require fluid intake. Since tablets require up to 200 ml of fluid intake, which is ~25% of a 7-year-old’s bladder capacity, the Melt formulation is more suited to the antidiuretic indication of desmopressin [40]. Good pharmacodynamic data are available for the Melt and its dosing in children with enuresis [54].

Medication should be taken 1 h before the last void before bedtime to allow timely enhanced concentration of urine to occur. Fluid intake should be reduced from 1 h before desmopressin administration and for 8 h subsequently to encourage optimal concentrating capacity and treatment response, as well as to reduce the risk of hyponatremia/water intoxication.

Desmopressin is only effective on the night of administration; therefore, it must be taken on a daily basis. Full adherence is required to avoid wet nights. Desmopressin acts immediately, but in our expert opinion, the initial duration of treatment should be for 2–6 weeks, to ascertain its anti-enuretic effect. If a sufficient degree of improvement is experienced, then treatment can be continued for an additional 3 months—where appropriate, country-specific regulations regarding treatment breaks should be followed. If patients are dry on treatment after this initial period, breaks are recommended to ascertain whether the problem has resolved and therapy is no longer necessary. If the child does not achieve complete dryness, or if wetting resumes once treatment is withdrawn, it should be continued/resumed. There is some evidence that structured withdrawal of medication may reduce relapse rates following its discontinuation [4, 29]. If a second voiding diary indicates nocturnal urinary production is not reduced, consider a dose increase (if maximum recommended dose has not been reached); otherwise, refer the child to a specialist.

Desmopressin is well tolerated, but clinicians should be aware that it is a potent antidiuretic and families must be educated regarding the rare possibility of patients developing hyponatremia/water intoxication with symptoms including headache, nausea, and vomiting. Self-titration of medication should be avoided.

## Enuresis alarm

Enuresis alarms have a level 1, grade A ICI recommendation [45]. The alarm is triggered when a sensor in the sheets or night clothes becomes wet, setting off an auditory signal causing the child to wake, cease voiding, and arise to void. Parents are advised to wake their child when the alarm is activated—otherwise, children are prone to turn it off and go back to sleep.

The alarm should be worn every night. Response is not immediate and treatment should be continued for 2–3 months or until the child is dry for 14 consecutive nights (whichever comes first). There may be cultural differences in its acceptability, as it may be highly disruptive for the household and may require a significant commitment of time and effort. The family must be motivated and adhere to this therapy if it is to be successful so they should be preemptively apprised of likely difficulties, but assured the first few weeks are the most troublesome. Doctors should monitor the child’s progress early to address any problems and facilitate adherence. The response rate is high in families who continue treatment for a sufficient period, with relatively low relapse rates (though lasting cure rate is still <50%) [19]. Poor compliance and early withdrawal from treatment are common [14, 56, 58], which may exacerbate parental intolerance. For these reasons, alarm treatment may not be suitable for some families; the clinician should exercise judgment as to whether alarm therapy is appropriate. In cases where the child or his or her family is reluctant to accept the alarm, desmopressin is the alternative [59].

## Importance of adherence to the management plan

It is estimated that ~30% of non-responders are not taking medication correctly [1, 50]. Non-adherence to recommendations regarding timing of medication, voiding before bedtime, and limitation of evening fluids can reduce treatment success [8]. Moreover, compliance is often overestimated, both by patients and MDs; therefore, it should be documented in a diary.

Patients who appear treatment-resistant should be advised of the importance of full adherence and asked if they have had any difficulty with complying with recommendations. It is important to cultivate an open atmosphere for discussion, since children may be reluctant to admit non-adherence if they feel their doctor is being judgmental.

## Non-response

If children show no improvement with one first-line treatment, despite adherence, an alternative therapy should be tried.

## Follow-up

Following successful treatment with either the alarm or desmopressin, patients should be advised to contact the clinic if relapse is experienced after discontinuation of therapy. If relapse occurs, further desmopressin, alarm, or combined therapy should be considered.

## Other strategies

Arousal training (where a rapid response to the alarm is rewarded) may be beneficial, as demonstrated in the original study [49, 52]. However, experience in clinical practice has had less than convincing results (resulting in a level 3, grade C recommendation from ICI); alarm alone is accepted best practice behavioral therapy.

A small number of alternative pharmacological approaches exist which may be considered if the patient is resistant to desmopressin and/or the alarm. Prescribing these should be restricted to specialized enuresis centers and not undertaken by MDs.

## Risk factors for treatment resistance and management strategies in these patients

The most likely fundamental reason for not responding to alarm or desmopressin therapy is that the actual diagnosis is NMNE and not MNE. When a detailed history is obtained, the majority of these children have at least subtle daytime symptoms [30]. Therefore, the importance of taking a good clinical history, as presented in our CMT (Table 1), is paramount. If a patient is treatment-resistant and a bladder diary has not been completed, it is imperative this is undertaken or to refer the child to a specialty center as OAB and dysfunctional voiding may be present.

The subjective hallmark of OAB during the daytime is urgency. However, children with OAB may compensate by drinking very little during the day, therefore masking this symptom [62]. If OAB is suspected, a bladder diary should be repeated with a standardized (e.g., 25–30 ml/kg/day) fluid intake. In some children, symptoms of OAB are truly not present while awake, although there is marked detrusor overactivity during sleep [62]. Children with severe OAB/reduced MVVs and those who are incontinent during the day may benefit from a period of bladder rehabilitation, such as scheduled voiding (with or without use of a programmable watch).

Children may have dysfunctional voiding (i.e., habitually contract the urethral sphincter during voiding), which is not identified by general screening for daytime symptoms. Dysfunctional voiding is common if there is recurrent urinary tract infection and constipation. If suspected and/or the patient is treatment-resistant, specialist referral for uroflowmetry and assessment of post-micturition residue is recommended. For children with suspected day and night detrusor overactivity, a combination of oxybutynin and desmopressin may be indicated (level 2, grade B).

Some patients with MNE may be resistant to desmopressin. This kind of patient is made of a heterogeneous group of subtypes and, as such, is beyond the scope of this approach; however, they can be generally subdivided into two subtypes—those with and without persistent NP.

Nocturnal urine volumes can give important information in the identification of patients with persistent NP. In patients with persistent NP, the following possibilities should be excluded before increasing the dose:

- Insufficient therapy compliance [50]
- No micturition before sleep
- Administration of drug <1 h before last void before sleep
- Excessive fluid intake after or during the hour before desmopressin administration [8]
- Diabetes insipidus (renal/central, partial)

If the problem persists despite efforts to correct it, the patient is desmopressin-resistant and should be referred. Desmopressin-resistant NP is a well-known clinical entity [34]; several pathogenetic factors play a key role, including abnormalities in maximal concentrating capacity, nocturnal osmosis, sodium and calcium excretion, nycthemeral rhythm of prostaglandins, blood pressure, glomerular filtration rate, calciuria, diet factors, and/or sleep pattern [10, 36, 38, 51]. Recording of nocturnal urine volume can be repeated during treatment to see whether output is reduced or not.

The dose of desmopressin needed to reach maximum concentrating capacity may vary between patients. Therefore, assuming a bladder diary has been completed and NMNE properly excluded, treatment resistance may be due to inadequate dosing. Caution is mandatory with dose escalation; if children do not void in the morning, the dose should not be increased as this is suggestive of prolonged bioactivity.

### Comorbidities associated with treatment resistance

Bowel habits are linked with lower urinary tract symptoms and urinary incontinence in particular. Constipation is a risk factor for enuresis and treatment resistance; it should be treated prior to devising a management plan for enuresis (see Box 3). Constipation is suspected in all patients with small volume fecal incontinence. Advice regarding sufficient fluid and dietary fiber intake, regular toilet habits (e.g., defecation every morning after breakfast), and potential use of laxatives should be provided.

Psychiatric conditions are additional risk factors for treatment resistance. There is an increasing number of reports regarding the beneficial effects of tricyclic antidepressants (e.g., imipramine) in selected subtypes of complicated and therapy-resistant children, such as those with attention deficit hyperactivity and sleep disorders. However, these subtypes need to be treated by specialists. Unless they are aware of, and know how to manage, its side effects, MDs should not prescribe imipramine, mainly because of its well-known potential for cardiotoxicity.

## Conclusions

Enuresis is a common condition that can be very upsetting and disruptive to family life. The child’s sleep architecture and cognition may be affected by repeated bladder signaling during the night. In many cases, MNE can be effectively treated by MDs through education that includes its causes and management, advice on eating, drinking and toileting habits, and prescription of appropriate treatments following a basic or enhanced evaluation strategy.

Our consensus opinion is based on the finding that evaluation (with/without voiding diaries) and an assessment of family preference and motivation can direct first-line treatment consisting of desmopressin (in particular the lyophilisate formulation) for patients with NP but without reduced MVV and an enuresis alarm for children with reduced MVV and normal nighttime urine output. Combination therapy (alarm + desmopressin) can also be considered for patients with both reduced MVV and NP or when children using an alarm awaken more than once during the night.

If one treatment does not work, another can be tried (assuming NMNE is thoroughly excluded using a bladder diary). The key message here is not to give up when attempting to devise an appropriate treatment strategy. If response cannot be achieved by the MD, with either strategy 1 or 2, the child should be referred to a specialist to allow all possible therapeutic avenues to be explored.
